# Efficient removal of hexavalent chromium from water by an adsorption–reduction mechanism with sandwiched nanocomposites†

**DOI:** 10.1039/c8ra01805g

**Published:** 2018-04-23

**Authors:** Weikang Liu, Liang Yang, Shihao Xu, Yao Chen, Bianhua Liu, Zhong Li, Changlong Jiang

**Affiliations:** School of Chemical Engineering, Anhui University of Science and Technology Huainan Anhui 232001 China zhongli@aust.edu.cn; Institute of Intelligent Machines, Chinese Academy of Sciences Hefei Anhui 230031 China cljiang@iim.ac.cn; State Key Laboratory of Transducer Technology, Chinese Academy of Sciences Hefei Anhui 230031 China

## Abstract

Hexavalent chromium Cr(vi), one of the most toxic contaminants, is released in the environment due to various anthropogenic activities. This study presents a novel sandwiched nanocomposite synthesized using graphene oxide (GO), manganese dioxide (MnO_2_) nanowires, iron oxide (Fe_3_O_4_) nanoparticles and polypyrrole (PPy) to remove hexavalent chromium ion Cr(vi) from water by an adsorption–reduction mechanism. In the sandwiched nanocomposites, GO provided enough surface area, functional groups, and hydrophilic surface for efficient absorption. Fe_3_O_4_ nanoparticles with excellent magnetic properties make it easy to separate and recover from water. Under acidic conditions, MnO_2_ nanowires act as both template and oxidant to initiate the polymerization of pyrrole monomers on its freshly activated surface to obtain GO/MnO_2_/Fe_3_O_4_/PPy (designated as GMFP) nanocomposite. GMFP could effectively adsorb Cr(vi) through electrostatic attraction, and the adsorbed Cr(vi) ions were partly reduced to trivalent chromium Cr(iii) (62%), resulting in the efficient adsorption and high removal of Cr(vi) from water. Hexavalent chromium adsorption by GMFP is strongly pH dependent and the adsorption kinetics followed the pseudo-second-order model. The Langmuir isothermal model described the adsorption isotherm data well and the maximum adsorption capacity was up to 374.53 mg g^−1^ at pH 2.0. These experimental results suggested that GMFP had great potential as an economic and efficient adsorbent of hexavalent chromium from wastewater, which has huge application potential.

## Abstract 2

A sandwiched nanocomposite has been developed to remove hexavalent chromium ion Cr(vi) from water by an adsorption–reduction mechanism.

## Graphical abstract

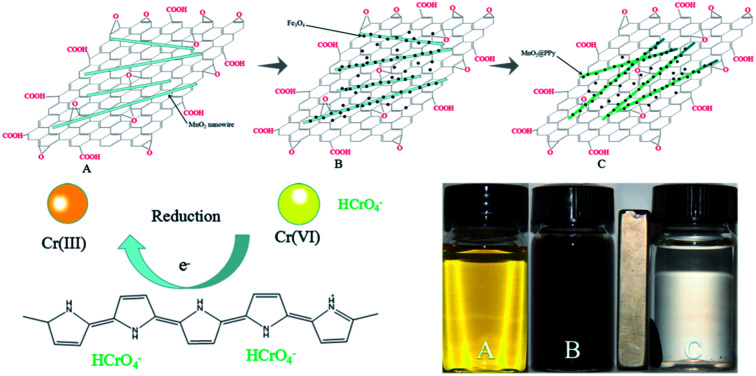

## 1. Introduction

The existence of heavy metal ions, such as Pb(ii), Fe(iii), Hg(ii), Cu(ii) and Cr(vi) in groundwater is one of the most serious environmental problems today.^1,2^ These toxic contaminants are a serious danger to human health. They enter the ecosystem through industrial and agricultural processes as well as mining activities.^3,4^ Among the toxic metal ions, chromium is a common contaminant in the environment coming from various anthropogenic activities, including metal electroplating, steelworks manufacturing, leather tanning, synthesis of pigments and so on. It is found that chromium exists in the environment in two stable states: trivalent Cr(iii) and hexavalent Cr(vi), and the toxicity of the hexavalent form is five hundred times greater than that of the trivalent form.^5^ Cr(vi) can cause many health problems, such as liver damage, pulmonary congestion, asthma, and severe diarrhea.^1,5^ Therefore, various agencies such as WHO and USEPA have given a tolerable limit of 0.05 mg L^−1^ for dissolved Cr(vi) in drinking water, and that for total chromium (all form of chromium) is 2 mg L^−1^.^6^ Therefore, it is very urgent to remove Cr(vi) from wastewater or reduce Cr(vi) to less toxic Cr(iii) prior to discharge into the environment. Until now, several methods have been reported to reduce the harmful effects of Cr(vi) such as electrical enrichment, reverse osmosis, ion exchange and adsorption.^7–10^ Owing to the simple procedure, cost-effective, and high efficiency, adsorption methods have great potential for the removal of Cr(vi) and thus attracting more and more attention in recent years.^11^

Different types of adsorbent materials have been widely used for the removal of Cr(vi) from wastewater. However, conventional adsorbents often show a limited adsorption capacity or not easily separated even cause potential secondary pollution because they do not have enough surface area, functional groups, and hydrophilic surface.^12^ Therefore, it is important to fabricate a nanomaterials with excellent performances of adsorption, reduction, and collectability simultaneously for the removal of Cr(vi) from wastewater.

Among the various materials, graphene oxide (GO), a two-dimensional carbon nanomaterial, has been used as a highly efficient adsorbent to remove heavy metal ions because of having an ultralarge specific surface area and abundant oxygen-containing groups (*e.g.* –OH and –COOH).^13–16^ And the idea of GO coated with MnO_2_ may be utilized in synthesizing new nanocomposite materials which would have better performances in metal ions adsorption applications. Polypyrrole (PPy) carries large amounts of positively charged nitrogen atoms in the polymer chains, which render it a good prospect in adsorption application.^17–19^ Although GO/MnO_2_/PPy has high adsorption and reduction abilities for Cr(vi), the resulting mixture could not be easily collected from water after treatment towards Cr(vi).^20^ Herein, Fe_3_O_4_ was incorporated with GO/MnO_2_/PPy to obtain GO/MnO_2_/Fe_3_O_4_/PPy, which displayed a high removal ability on Cr(vi) through adsorption and reduction, and an excellent magnetic collectability from aqueous solution. The optimal condition and the removal mechanism were investigated. This work provides a facile, efficient, and environmentally-friendly approach for the remediation of Cr(vi)-contaminated wastewater.

## 2. Results and discussion

### 2.1. Characterization

**Scheme 1:**
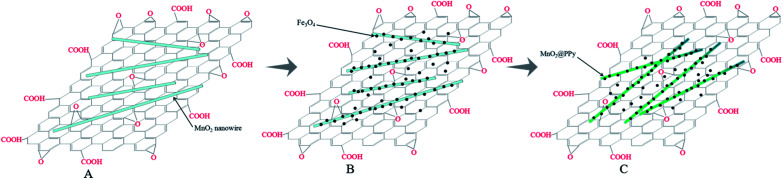
Schematic illustration of the formation mechanism of (A) GO/MnO_2_, (B) GO/MnO_2_/Fe_3_O_4_, (C) GO/MnO_2_/Fe_3_O_4_/PPy.

Aim to design materials with high adsorption capacity for the chromium removal, a sandwiched nanocomposite has been developed with utilizing graphene oxide (GO), manganese dioxide (MnO_2_) nanowires, iron oxide (Fe_3_O_4_) nanoparticles and polypyrrole (PPy), as illustrated in Scheme 1. In this study, we employed GO as the substrate for its ultralarge specific surface area and abundant oxygen-containing groups. By means of hydrothermal method, the MnO_2_ nanowires were deposited at the surface of graphene oxide sheets. Fe_3_O_4_ was incorporated with GO/MnO_2_ to obtain GO/MnO_2_/Fe_3_O_4_, which displayed an excellent magnetic collectability as adsorbent for Cr(vi) remove from aqueous solution. Finally, by using pre-prepared MnO_2_ nanowires as the reactive templates, pyrrole monomers transport to the surface of MnO_2_ nanowires, leading to the formation of GO/MnO_2_/Fe_3_O_4_/PPy, which displayed a high removal ability on Cr(vi) through adsorption and reduction.

**Fig. 1:**
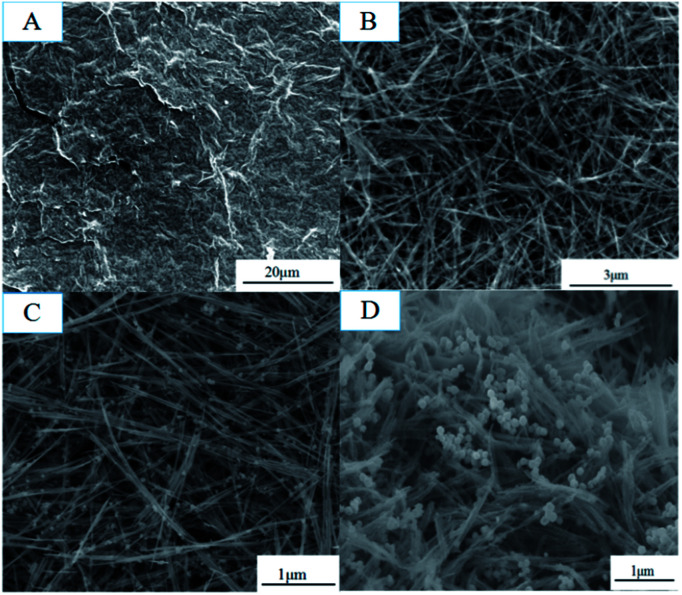
SEM images of (A) GO, (B) GO/MnO_2_, (C) GO/MnO_2_/Fe_3_O_4_, and (D) GMFP nanocomposites.

The obtained GMFP nanocomposite was first characterized by scanning electron microscopy (SEM) (Fig. 1). GO was prepared by modified Hummers method from natural flake graphite (Fig. 1A). Then GO/MnO_2_ nanomaterials were fabricated *via* hydro-thermal method. As shown in Fig. 1B, the MnO_2_ component in the composite is nanowire, which is several hundred nanometers to several micrometers in length and homo-geneously and densely attached on the surface of graphene sheets. Fig. 1C is the GO/MnO_2_/Fe_3_O_4_ nanocomposites, in which the Fe_3_O_4_ nanoparticles were well distributed on the surface of GO/MnO_2_ nanocomposites. After functionalized with pyrrole, as displayed in Fig. 1D, the resulting sample has rough surface. MnO_2_ is a strong oxidant and acts as chemical oxidative initiator for pyrrole polymerization. Since the MnO_2_ serves as oxidant and template in the chemical reaction, PPy shell is coated on MnO_2_ nanowires to form core–shell structure.

**Scheme 2:**
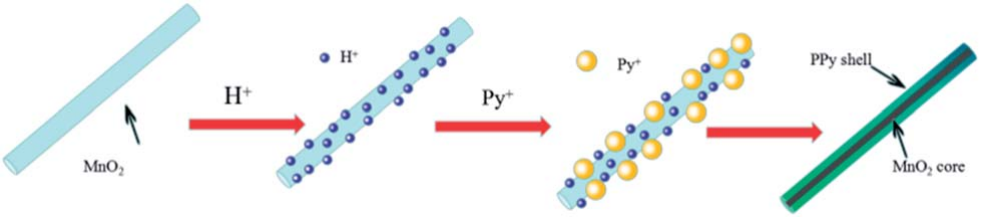
Schematic illustration of the formation mechanism of the MnO_2_/PPy nanocomposites in acid condition.

**Fig. 2:**
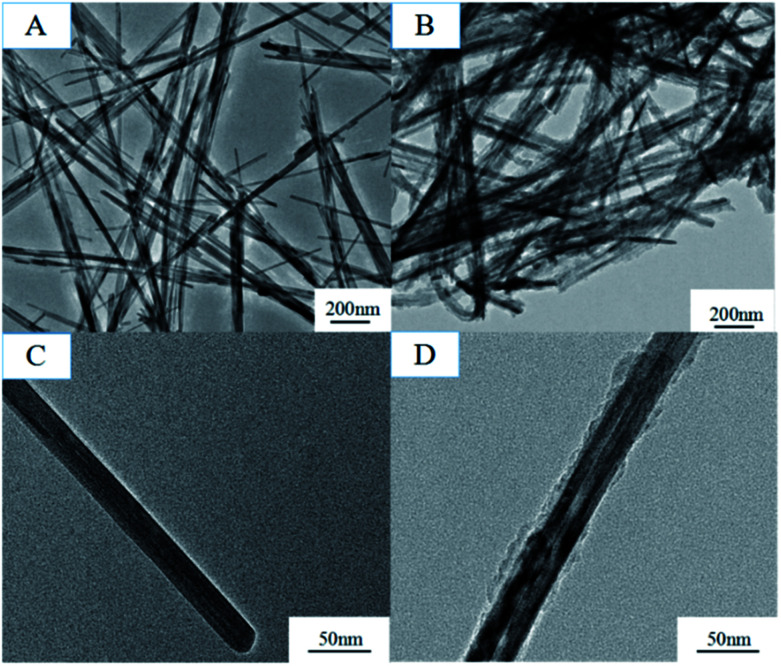
TEM images of (A) and (C) MnO_2_, (B) and (D) MnO_2_/PPy.

The formation mechanism of MnO_2_/PPy coaxial nanowires is illustrated in Scheme 2. It is understandable that the core of the coaxial nanowires is α-MnO_2_ and the shell is PPy. In our case, the α-MnO_2_ nanowire surface exposed in acidic solution contacts with pyrrole monomers to proceed with a redox reaction. When H^+^ ions are added in the solution, some of them are adsorbed on the surface of α-MnO_2_ nanowires due to the electrostatic attraction. After adding pyrrole, the monomers transport to the surface of α-MnO_2_ nanowires because of their intrinsic hydrophilic characteristic in acid solution and polymerize over there, leading to the formation of PPy shell. Fig. 2 shows the TEM images of α-MnO_2_ nanowires and MnO_2_/PPy coaxial nanowires. As shown in Fig. 2A, the MnO_2_ sample demonstrates 1-D nanostructured crystals, no other morphologies are observed in the sample. TEM image in Fig. 2C further confirms the formation of MnO_2_ nanowires with average thickness of 22.52 nm and average length of several microns. Fig. 2B shows the TEM images of MnO_2_/PPy coaxial nanowires, the average thickness of the MnO_2_/PPy coaxial nanowires was 49.38 nm. So the average thickness has changed greatly before and after the treatment of PPy. TEM image in Fig. 2D clearly reveals the core/shell morphology of the MnO_2_/PPy nanowires with the outer layer of PPy with thickness about 5–10 nm and the inner layer of MnO_2_ nanowires with the wall thinned, confirming the successful preparation of MnO_2_/PPy coaxial nanowires. The image of size distribution was shown in Fig. S1.†

**Fig. 3:**
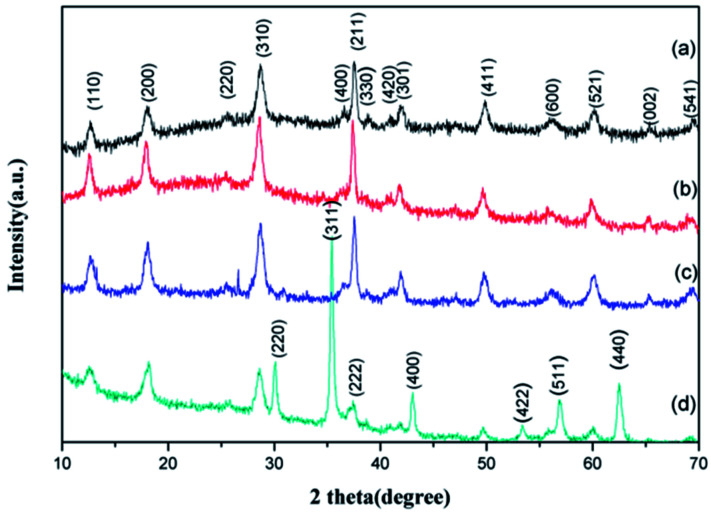
X-ray diffraction patterns of (a) MnO_2_, (b) MnO_2_/PPy, (c) GO/MnO_2_, (d) GMFP.

The crystal structure of the GMFP nanocomposite has been identified by X-ray power diffraction measurement. It can be clearly seen that diffraction peaks located at 2*θ* = 12.8, 18.1, 28.7, 36.5, 37.5, 42.0, 49.8, 56.1, 60.1, 65.6 and 69.5°, which can be assigned to the (110), (200), (310), (400), (211), (301), (411), (600), (521), (002) and (541) planes of α-MnO_2_ (JCPDS no. 44-0141) (Fig. 3a).^21,22^ No characteristic impurity peaks are observed, indicating the high purity of α-MnO_2_ nanowires. And seen from the XRD pattern of the MnO_2_/PPy in Fig. 3b, all diffraction peaks are similar to the pristine α-MnO_2_, confirming the presence of α-MnO_2_ in the composites after *in situ* polymerization. As shown in Fig. 3d, the diffraction peaks (2*θ* = 30.3°, 35.62°, 43.3°, 53.34°, 57.16°, and 62.76°) of Fe_3_O_4_ (JCPDS no. 75-0033) appeared in the XRD pattern of GMFP, suggesting that Fe_3_O_4_ was successfully combined with GO/MnO_2_.^23^

**Fig. 4:**
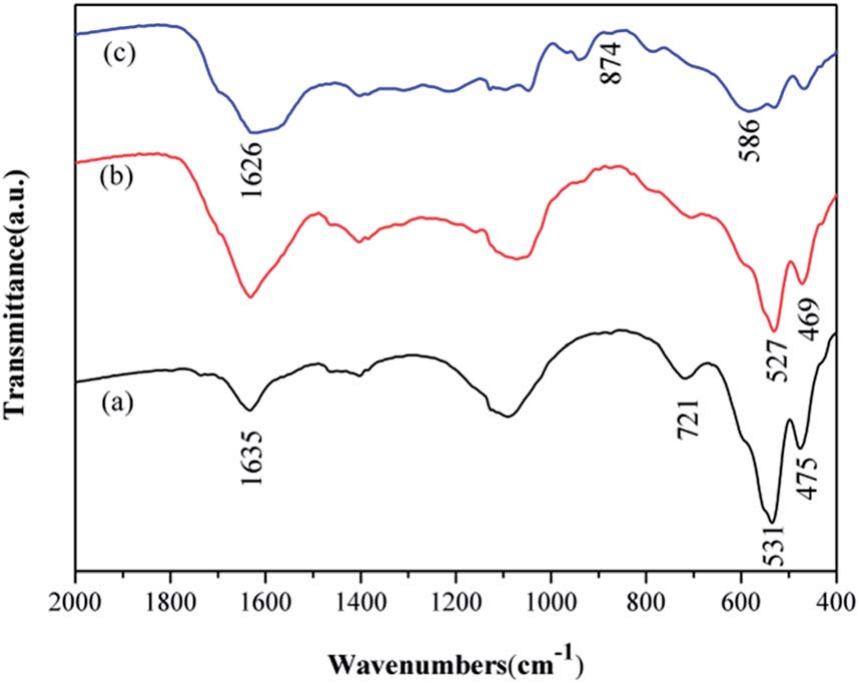
FT-IR spectra of (a) MnO_2_, (b) MnO_2_/PPy, (c) GMFP nanocomposites.

The structural information and chemical component of are also identified by the FT-IR spectroscopy. For comparison, the spectra for both α-MnO_2_ and MnO_2_/PPy coaxial nanowires are recorded and shown in Fig. 4. It can be seen that from Fig. 4a, the characteristic peaks of α-MnO_2_ appear at about 721, 531 and 475 cm^−1^, belonging to Mn–O vibrations of MnO_6_ octahedra in α-MnO_2_ nanowires, and at 1635 cm^−1^, relating to O–H vibrational mode of absorbed water. By contrast, in the spectrum of MnO_2_/PPy coaxial nanowires (Fig. 4b), the aforesaid peaks at 531 and 475 cm^−1^ shift to 527 and 469 cm^−1^, respectively, and all display attenuation in intensity, reflecting a mutual interaction between PPy and MnO_2_ that, most likely, is a hydrogen bond formed between oxygen atom of Mn–O and hydrogen atom of N–H in PPy.^24^ Such spectral information suggests that the PPy shell has been coated closely on MnO_2_ surface and in doping state. In Fig. 4c and a characteristic peak at 586 cm^−1^ for the Fe–O stretching vibration of Fe_3_O_4_ appeared. While the transmissions around 1626 and 874 cm^−1^ in Fig. 4c from the amine-functionalized nanocrystals matched well with that from free 1,6-hexa-diamine, indicating the existence of the free –NH_2_ group on the amine-functionalized Fe_3_O_4_ nano-materials.

### 2.2. Determination of Cr^6+^ concentration

The concentration of Cr^6+^ was analyzed by UV spectro-photometric method,^25^ and the results were shown in Fig. S2 and S3 (ESI†). When absorbance is plotted against concentration, the data are regressed linearly, so we could calculate concentration of hexavalent chromium ion from the absorbance.

### 2.3. Effect of solution pH

The solution pH has been acknowledged as the most important factor governing metal adsorption onto the adsorbent.^26^ To investigate the adsorption capacity of GMFP under both acidic and alkalic conditions, pH values from 2 to 12 at initial concentrations of 300 mg L^−1^ to evaluate the effect of pH on the Cr^6+^ adsorption process.

**Fig. 5:**
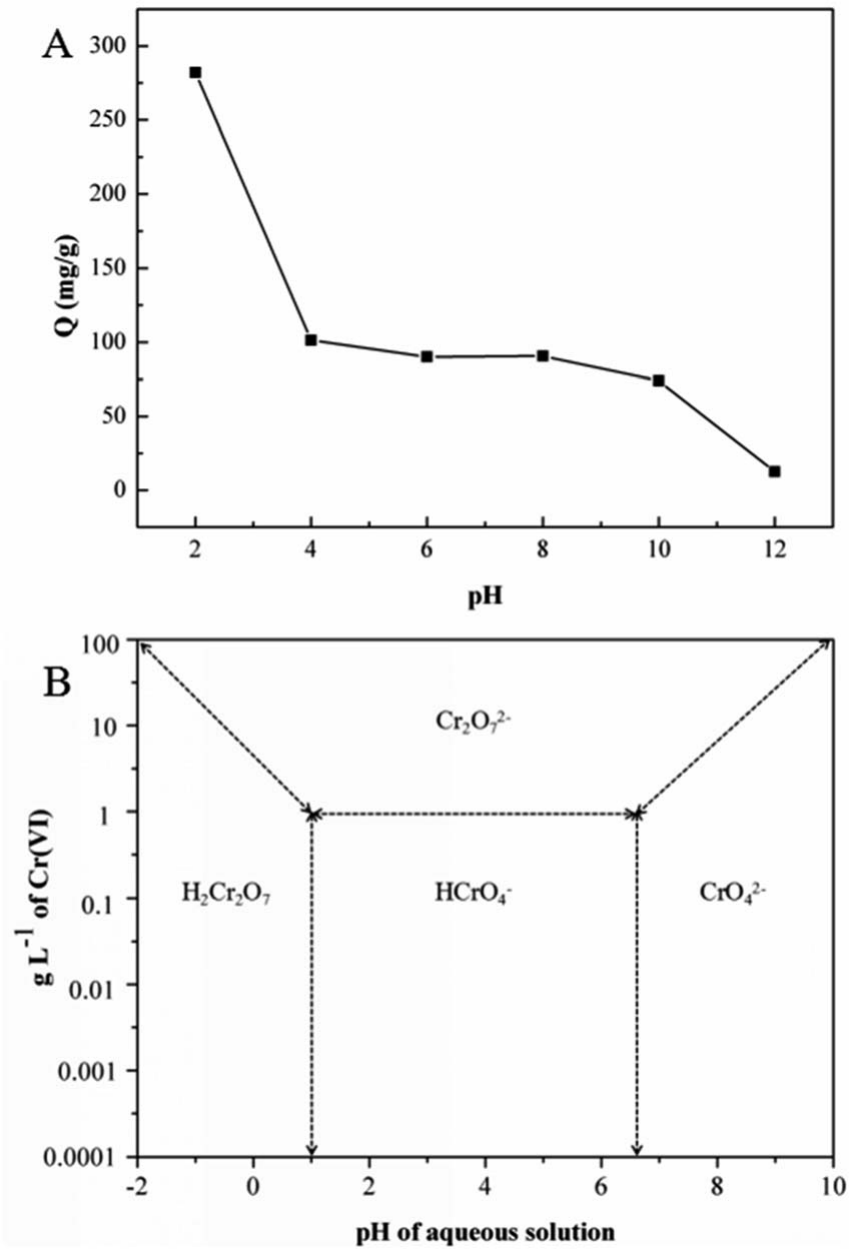
(A) Effect of pH on the adsorption of Cr(vi) by GMFP: temperature, 298.15 K; adsorption time, 6 h; and initial Cr(vi) concentration, 300 mg L^−1^, (B) relative distribution of Cr(vi) species in water as a function of pH and Cr(vi) concentration (adapted from ref. 1).

Solution pH affects both the surface charge of an adsorbent and the speciation of metal ions. The effect of initial solution pH on Cr(vi) adsorption by the GMFP composite was therefore studied and illustrated in Fig. 5. As shown in Fig. 5A, the Cr(vi) adsorption capacity decreased as the solution pH increasing from 2 to 12. Cr(vi) speciation in solution is known to be highly pH dependent (Fig. 5B). Chromic acid (H_2_CrO_4_) occurs when pH is less than 1. Furthermore, from acidic pH 1 to the neutral pH 7, the hydrogen chromate ion (HCrO_4_^−^) exist, whereas, above the neutral pH, only chromate ions (CrO_4_^2−^) exist in the solution.^27,28^ At a lower pH, the adsorption effect is high because predominant Cr^6+^ species mainly exists in monovalent HCrO_4_^−^ form, which is then gradually converted to divalent CrO_4_^2−^ and Cr_2_O_7_^2−^ as pH increases. The adsorption free energy of HCrO_4_^−^ is lower than that of CrO_4_^2−^ and Cr_2_O_7_^2−^; and consequently, HCrO_4_^−^ is more favorably adsorbed than CrO_4_^2−^ and Cr_2_O_7_^2−^ at the same concentration. As the pH increases, the GMFP nanocomposites surface becomes increasingly deprotonated so that the amount of positive surface charges is significantly decreased, leading to a reduction in the adsorption capacity of Cr^6+^. Thus, the adsorption quantities of Cr^6+^ at a lower pH are larger than that of at higher pH. The controlled experiments about without use of adsorbent in the presence of acid and without acid only use adsorbent have been made. The result was shown in Fig. S4.† The figure shows that the concentration of Cr(vi) does not change without use of adsorbent in the presence of acid (pH = 2), and the Cr(vi) percentage removal is about 26% without acid only use the adsorbent. This result shows that the pH value is an important factor affecting the adsorption efficiency.

### 2.4. Adsorption isotherms

Batch equilibrium adsorption experiments were used for adsorption assessment through plots of adsorption isotherms. The maximum adsorption capacity can be obtained from adsorption isotherms. Adsorption isotherm consists of two important parameters, Langmuir and Freundlich isotherms.

Langmuir adsorption isotherms have been successfully applied to many real adsorption processes. A basic assumption of Langmuir theory is that adsorption takes place at specific homogeneous sites within the adsorbent. It is assumed that once a molecule occupies a site, no further adsorption can occur at that site. Theoretically, a saturation value is reached and no further sorption can occur. A linear form of this expression is:
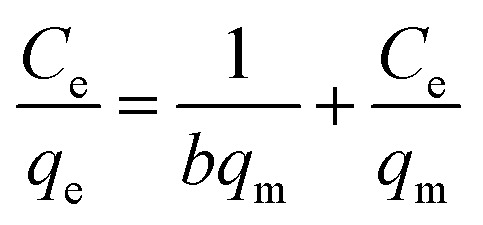
where *q*_e_ (mg g^−1^) is the amount of solution adsorbed per unit mass of the adsorbent, *C*_e_ (mg L^−1^) is the solute equilibrium concentration, *q*_m_ (mg g^−1^) is the maximum adsorbate amount that forms a complete monolayer on the surface, and *b* (L mg^−1^) is the Langmuir constant related to adsorption heat. When *C*_e_/*q*_e_ is plotted against *C*_e_ and the data are regressed linearly, the *q*_m_ and *b* constants can be calculated from the slope and the intercept.

**Fig. 6:**
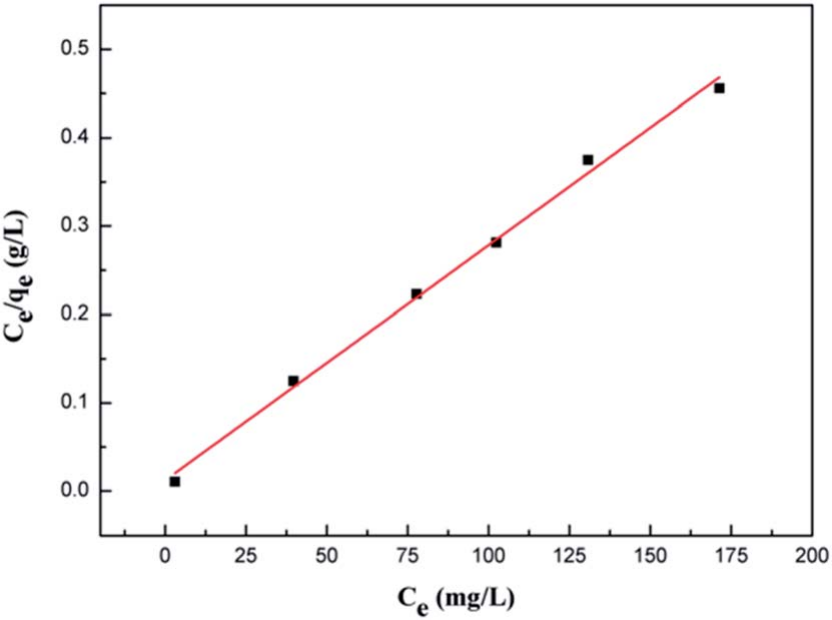
Fit of equilibrium data to Langmuir isotherm model.

The values of these parameters, as analyzed from the plots shown in Fig. 6, are summarized in Table 1. According to the obtained results, the adsorption data of the Cr(vi) ions of GMFP nanocomposites were fitted particularly well with the Langmuir model, with good correlation coefficients. The maximal adsorption capacity of Cr(vi) ions of GMFP nano-composites was about 374.53 mg g^−1^. By comparison to the reported adsorbents (Table 2), GMFP has a superb removal capacity toward Cr(vi). Therefore, GMFP is a potential material for Cr-contaminated wastewater cleanup.

**Table 1:** Parameters of the Langmuir isotherms for Cr^6+^ adsorption onto GMFP nanocomposites

pH	*T* (K)	*q*_m_ (mg g^−1^)	*b*	*R* ^2^
2	308.5	374.53	0.320	0.995

**Table 2:** Comparison of maximum adsorption capacity of Cr(vi) on GMFP with other adsorbents

Absorbent	*Q*_m_ (mg g^−1^)	Optimum pH	Ref.
Graphene oxide	65.2	2	29
GO/MnO_2_/Fe_3_O_4_	193.1	2	5
MnO_2_/Fe_3_O_4_/o-MWCNTs	186.9	2	30
PPy nanoclusters	180.4	5	31
PPy/Fe_3_O_4_	169.4	2	32
PPy/o-MWCNTs	294.1	2	33
PPy/2,5-Diaminobenzene	222.2	2	34
PPy/sepiolite nanofibers	302	2	35
GO/MnO_2_/Fe_3_O_4_/PPy	374.5	2	This study

### 2.5. Adsorption kinetics

**Fig. 7:**
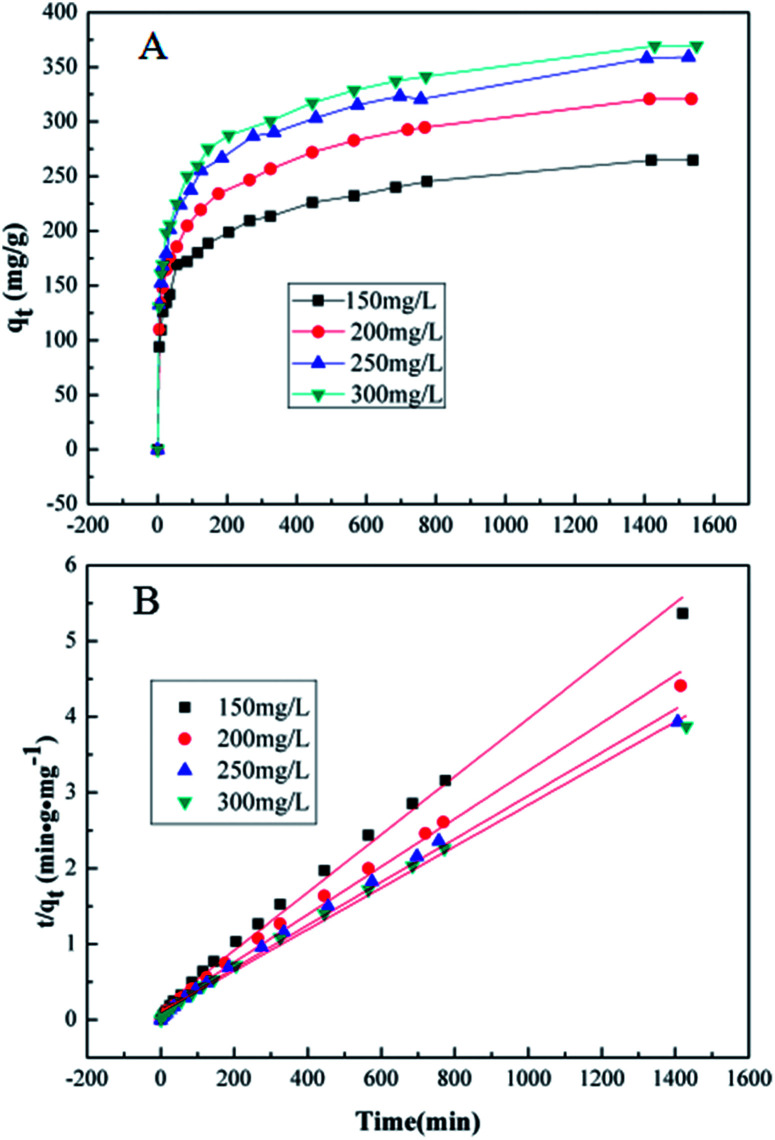
(A) The adsorption capacity of GMFP nano-composites for different concentrations of Cr(vi) ions with time. (B) The pseudo-second-order model for adsorption of Cr(vi) ions by GMFP nanocomposites.

Adsorption kinetics, demonstrating the solute uptake rate, is one of the most important factors which represent the adsorption efficiency of the GMFP and therefore, determines their potential applications. The effect of adsorption time on the adsorption capacity at different initial solution concentrations is shown in Fig. 7A. The removal of Cr^6+^ increases with increasing contact time. To better understand the adsorption behaviors, the kinetic adsorption data were simulated with the pseudo-second-order rate equation, which are expressed as follows:
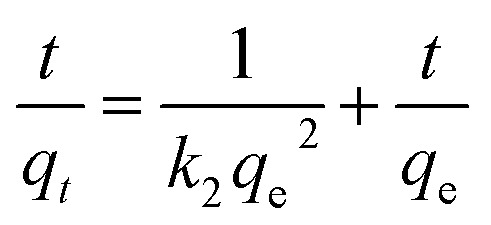
where *q*_e_ and *q*_*t*_ (mg g^−1^) are the adsorption capacities at equilibrium and at time *t* (min), *k*_2_ (g mg^−1^ min^−1^) are the pseudo-second-order rate adsorption constants.

The values of these parameters, as analyzed from the plots shown in Fig. 7, are summarized in Table 3. It is found that the removal of Cr^6+^ increases with increasing contact time. The equilibrium capacity *q*_e_ calculated from the pseudo-second-order kinetic model were 261.78, 316.45, 350.87 and 364.96 mg g^−1^ for the 150, 200, 250 and 300 mg L^−1^ initial Cr^6+^ solution. The rate constants (*k*_2_) were very small, suggesting that the adsorption process was not very fast. Fig. 7B and Table 3 show the pseudo-second-order model for adsorption of Cr(vi) by GMFP nanocomposites. From the plots *t*/*q*_*t*_*vs. T* of GMFP nanocomposites adsorbent at initial concentrations of Cr(vi) varied from 150 to 300 mg L^−1^, the pseudo-second-order rate constant (*k*_2_) decreases from 9.588 × 10^−5^ to 7.396 × 10^−5^ g mg^−1^ min^−1^. The calculated *q*_e_ is also close to the theoretical one, and the correlation coefficient (*R*^2^) is above 0.994. This result indicates that the adsorption kinetics of the Cr(vi) removal by the GMFP nanocomposites follows the pseudo-second-order model, suggesting it is a chemisorption process.

**Table 3:** Kinetics parameters for Cr(vi) adsorption onto GMFP nanocomposites with different concentrations

Concentration of Cr(vi) ions (mg L^−1^)	*q*_e_ (mg g^−1^)	*k*_2_ (g mg^−1^ min^−1^)	*R* ^2^
150	261.78	9.588 × 10^−5^	0.9943
200	316.45	7.856 × 10^−5^	0.9941
250	350.87	7.454 × 10^−5^	0.9936
300	364.96	7.396 × 10^−5^	0.9955

### 2.6. Mechanisms of adsorption

**Scheme 3:**
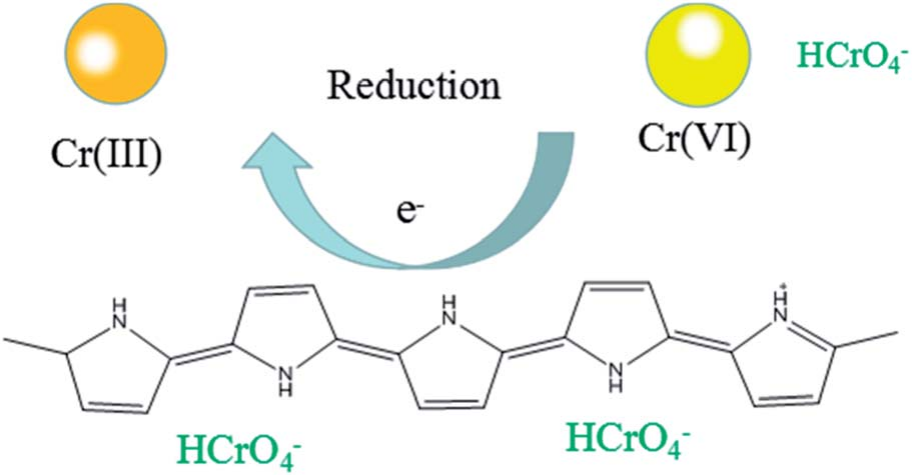
Schemes illustrations of mechanism on the removal of Cr(vi).

We also use the XPS spectra to investigate the mechanism of Cr(vi) adsorption with the GMFP nanomaterials in current system (see Fig. S5†). The presence of the elements C, N, O, Mn and Fe with high contents in the GMFP was evidenced by the photoelectron lines of the wide-scan XPS spectrum at 285, 401, 532, 641 and 711 eV, attributed to C 1s, N 1s, O 1s, Mn 2p and Fe 2p, respectively (Fig. S5A†). The Mn 2p XPS spectrum exhibits two characteristic peaks at 642.0 and 653.5 eV, corresponding to the Mn 2p_3/2_ and Mn 2p_1/2_ spin–orbit peaks of α-MnO_2_, further confirming the presence of α-MnO_2_ in the composite (Fig. S5B†). In the high-resolution Fe 2p XPS spectrum (Fig. S5C†), the peaks of Fe 2p_3/2_ and Fe 2p_1/2_ at 710.6 and 724.1 eV were the characteristic positions of Fe_3_O_4_,^36^ indicating the existence of Fe_3_O_4_ nanoparticles in the MnO_2_ support. In the formation process of the GMFP nanocomposites, the initial GO was reduced to graphene, confirmed by significantly improving the intensity of sp^2^ C–C bonds of graphene and decreasing the oxygen containing carbon (epoxy C–O, carbonyl C=O, and carboxyl) (Fig. S5D†). XPS spectra of the GMFP nanocomposites before and after adsorption of Cr(vi) were shown in Fig. S5E.† Before adsorption of Cr(vi), no Cr ions signals are observed in the XPS spectrum of GMFP. However, two energy bands at about 577.3 and 586.8 eV appear after adsorption of Cr(vi), corresponding to the binding energies of Cr 2p_3/2_ and Cr 2p_1/2_. This observation suggests the existence of both Cr(iii) and Cr(vi) on the surface of the GMFP after their adsorption of Cr(vi). The existence of Cr(vi) species on the surface of GMFP can be attributed to the adsorption of Cr(vi) ions through the anion exchange property of the surface of GMFP under acidic conditions. However, the appearance of Cr(iii) on the surface of GMFP indicates that some fraction of adsorbed Cr(vi) was reduced to Cr(iii) during the adsorption process. After treatment of Cr(vi), the N 1s peak of GMFP shifted to a lower binding energy (Fig. S5F†), and the peak width of N 1s decreased significantly, suggesting that the presence of positive nitrogen group in polypyrrole was regarded as a great contribution to the reduction process.^37,38^ There could be a possibility of reduction of Cr(vi) to Cr(iii) in the presence of functional groups such as –OH, –COOH, and –NH–, on the surface of GMFP at low pH values due the occurrence of redox reactions between the surface groups and the Cr(vi). The whole process was represented by the following eqn and Scheme 3:

1 GMFP + Cr_2_O_7_^2−^ + H^+^ → Cr^3+^ + HCrO_4_^−^ + H_2_O

2 HCrO_4_^−^ + 7H^+^ + 3e → Cr^3+^ + 4H_2_O

### 2.7. Desorption performance of the GMFP

For practical application, recycling and regeneration of the adsorbent is indispensable because the better repeated availability of advanced adsorbents may reduce the overall cost of the adsorbent. Given that the adsorption of Cr^6+^ ions onto the GMFP is pH-dependent and that the lower pH is beneficial for the Cr^6+^ adsorption, the desorption of Cr^6+^ ions from the adsorbent can be achieved by increasing the system pH values. Therefore, for the reusability study, experiments were conducted at alkaline condition. It can be seen that the GMFP nanocomposite still possessed more than 282 mg g^−1^ of the adsorption capacities for Cr^6+^ after four cycles of reuse, indicating that GMFP have a good reusability for Cr^6+^ adsorption, which slightly decreased to 264 mg g^−1^ at the fifth cycle, reflecting the high adsorption and stability of GMFP (Fig. S4†).

### 2.8. Effect of co-existing ions

**Fig. 8:**
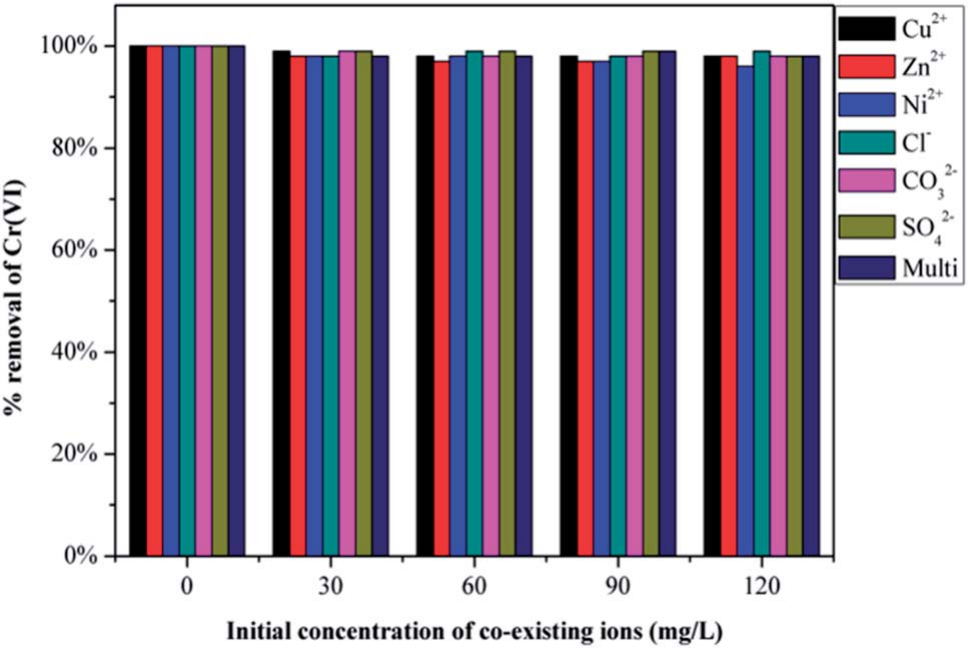
Effect of co-existing ions on the removal of Cr(vi) by GMFP.

Chromium-containing industrial wastewater also contains other types of particles, such as Cu^2+^, Zn^2+^, Ni^2+^, Cl^−^, CO_3_^2−^ and SO_4_^2−^. Therefore, it is essential to investigate the competitive influence of these co-existing ions on Cr(vi) removal and the results are presented in Fig. 8. Initially Cr(vi) removal was recorded using 50 mL of 200 mg L^−1^ of Cr(vi) solution and 30 mg of GMFP. The figure shows that both anions and cations in solution at varying concentrations do not significantly affect the removal of Cr(vi) by GMFP. These results can be explained by the surface properties of GMFP. At low pH, cations in solution are repelled from the positively charged surface of GMFP, and therefore, do not affect Cr(vi) removal. Anions such as Cl^−^, CO_3_^2−^ and SO_4_^2−^ are expected to compete with Cr(vi) because they are negatively charged (HCrO_4_^−^). But it is not observed as seen from Fig. 8. One possible reason is that Cl^−^, CO_3_^2−^ and SO_4_^2−^ are weaker oxidising agents than HCrO_4_^−^, they are not reduced by GMFP and therefore do not affect Cr(vi) adsorption.^33^

## 3. Conclusion

Hexavalent chromium is one of the extremely toxic heavy metals of great concern in water supplies. Until now, different types of adsorbent materials have been widely studied for the removal of Cr(vi) from wastewater. However, conventional adsorbents often show a limited adsorption capacity or difficult to collected from wastewater, these shortages limit these materials in actual application. For conducting polymers, polypyrrole (PPy) has been studied for the removal of Cr(vi) from wastewater because of its tunable morphology, excellent redox property and long-term environment stability. In this study, we present a magnetic nanocomposite named GMFP, was fabricated based on GO, MnO_2_, Fe_3_O_4_ and PPy, and it was confirmed by SEM, TEM, XRD, and FTIR. The MnO_2_/PPy composites with core/shell nanostructure were synthesized successfully *via* a simple approach, and performed excellently towards Cr(vi) adsorption in the aqueous solution, displaying high adsorption capacity (374.53 mg g^−1^) under acidic conditions. The results of XPS shows the mechanisms of Cr removal, its suggested the adsorption effect was mainly because of the electrostatic attraction between the surface of the bare GMFP and Cr(vi), and the reduction from Cr(vi) to Cr(iii) was mainly attributed to the functional groups (such as –OH, –COOH, and –NH–) of GMFP. This finding implicated that GMFP had great potential as an economical and efficient adsorbent of Cr(vi) from wastewater.

## Conflicts of interest

There are no conflicts to declare.

## Supplementary Material

RA-008-C8RA01805G-s001
