# Supplementary material for: Efficient removal of hexavalent chromium from water by an adsorption–reduction mechanism with sandwiched nanocomposites
Source: RSC Adv. 2018 Apr 23;8(27):15087–93. doi: 10.1039/c8ra01805g (PMC9080019; doi:10.1039/c8ra01805g)
Supplement: RA-008-C8RA01805G-s001 [file RA-008-C8RA01805G-s001.pdf]

Electronic Supplementary Material (ESI) for RSC Advances.
This journal is © The Royal Society of Chemistry 2018

Supplementary Information For

Efficient Removal of Hexavalent Chromium from Water by an Adsorption-Reduction Mechanism with Sandwiched Nanocomposites

Weikang Liu,[a] Liang Yang,[bc] Shihao Xu,[bc] Yao Chen,[bc] Bianhua Liu,[bc] Zhong Li[a] and Changlong Jiang*[bc]*

[a]School of Chemical Engineering, Anhui University of Science and Technology, Huainan, Anhui 232001, China.

[b]CAS Center for Excellence in Nanoscience, Institute of Intelligent Machines, Chinese Academy of Sciences, Hefei, Anhui 230031, China.

[c]State Key Laboratory of Transducer Technology Chinese Academy of Sciences, Hefei, Anhui 230031, China.

Experiment Section

Materials. Natural graphite flakes was supplied by Sigma Chemical Corporation. Potassium permanganate powder ($KMnO_4$, 99.5%), potassium persulfate ($K_2S_2O_8$, 99.5%), phosphorus pentoxide (P_2O_5, 98.0%), sulfuric acid (H_2SO_4, 96-98%), hydrochloric acid (HCl, 36-38%) and hydrogen peroxide aqueous solution (H_2O_2, 30 wt%) were purchased from Shanghai Chemical Reagent Corporation. Pyrrole of analytical grade was purchased from Aldrich Chemical Corporation. Aqueous solutions were all prepared using ultrapure water (18.2 MΩ cm) from a Millipore water purification system. All chemicals were used directly without any further purification in the experiments.

Synthesis of GO/MnO_2 composites. GO was synthesized by modified Hummers method. GO/MnO_2 nanowires were synthesized by a simple one-pot method. In brief, 0.75 g of $KMnO_4$ powder,0.1 g GO and 1.3 g $K_2S_2O_8$ were dissolved in 15 mL deionized water under ultrasonic condition for 40 min. This solution was then transferred into a Teflonlined autoclave and reacted at 140℃ for 24 h.

Synthesis of GO/MnO_2/Fe_3O_4 nanocomposites. The amine-functionalized magnetic nanoparticle was synthesized as previous work. Then 150 mg of GO/MnO_2 nanocomposites and 30 mg of amine-functionalized Fe_3O_4 nanoparticles were dissolved in 125 mL deionized water under ultrasonic condition for 40 min. After stirring at 35 °C for 3 h, the resulting system was filtered and washed several times using deionized water and ethanol absolute, and then dried at 45℃ for 10 h before use.

Synthesis of GMFP nanocomposites. 150 mg of the as-prepared GO/MnO_2/Fe_3O_4 nanocomposites were dispersed in 60 mL 0.01 M H_2SO_4 aqueous solution under ultrasonic condition and then cooled in an ice bath with a slow stir. 100 μL pyrrole monomers were dissolved in 40 mL 0.01 M H_2SO_4 aqueous solution. These two newly prepared solutions were rapidly mixed and then stirred in an ice bath for 3 h. The generated black product was filtered, then washed with deionized water and ethanol several times, and finally dried at 45℃ for 10 h.

Determination of Cr^{6+} concentration. The concentration of Cr^{6+} was analyzed by 1,5-diphenylcarbohydrazide spectrophotometric method. In each Cr^{6+} measurement experiment, a 0.2 mL solution containing Cr^{6+} was transferred into a 100 mL flask. Approximately 50 mL of deionized water, 0.5 mL of H_2SO_4 solution (50%), 0.5 mL of H_3PO_4 solution (50%), 2 mL of 1,5-diphenylcarbohydrazide solution were then successively added into the Cr^{6+} solution. The

mixture was forcefully shaken for 1 min and collected in a glass cuvette after being left to standing for 5 min. The resultant sample solution was transferred to the spectrophotometer for analysis at 540 nm.

Adsorption experiments. Analytical grade potassium dichromate was used to prepare a 1000 mg L^{-1} stock solution, which was further diluted to the required concentration before use. Different samples (0.01 g) of the adsorbents were added to 20 mL of Cr^{6+} solutions and being shaken for 24 h, the solid and liquid were separated by filtration using a 0.22 μm microporous membrane filter. And the residual concentration of Cr(VI) was measured by the UV spectrophotometric method.[24] The influence of the initial Cr(VI) concentration, pH, contact time, and temperature on the removal efficiency of Cr(VI) were investigated. After that, the adsorption capacity of Cr(VI) ions could be calculated by the following equation :

$$q_e = \frac{V(C_0 - C_e)}{m} \quad (1)$$

where q_e is the adsorbent adsorption capacity (mg g^{-1}), V is the volume of the solution (L), C_0 is the Cr^{6+} concentration (mg L^{-1}), C_e is the equilibrium Cr^{6+} concentration (mg L^{-1}), m is the weight of the adsorbents (g), and C_0 and C_e are the initial and equilibrium concentrations of Cr(VI) .

Desorption and regeneration experiments. To investigate the regenerability and reusability of GMFP nanocomposites, desorption experiments were conducted in a batch mode. After adsorption, desorption was carried out by washing the adsorbents with distilled water several times, and then the Cr^{6+}-adsorbed adsorbent were immersed in 100 mL of 0.5 M NaOH by shaking for 10 h. Before the second adsorption, the adsorbent was treated with 2 M HCl solution

and shaking for 5 h. The solid and liquid phases were separated by a magnet. The adsorption-desorption cycle was repeated six times using the same affinity adsorbents.

Characterization. The surface morphology and microstructure of samples were measured by scanning electron microscope (SEM) (Sirion 200, FEI Co.,USA) and transmission electron microscope (TEM) (Hitachi Co., Japan).The structure and composition analyses were conducted using a TTRIII X-ray diffractometer (XRD, Rigaku Co., Japan), a Fourier transform infrared (FTIR) spectrometer (iS10, Nicolet Co., USA), and an X-ray photoelectron spectroscope (XPS, ESCALAB 250, Thermo-VG Scientific Co., USA). The concentration of Cr(VI) ion was measured using a UV-vis spectrophotometer (UV-2550, Shimadzu Co., Japan).

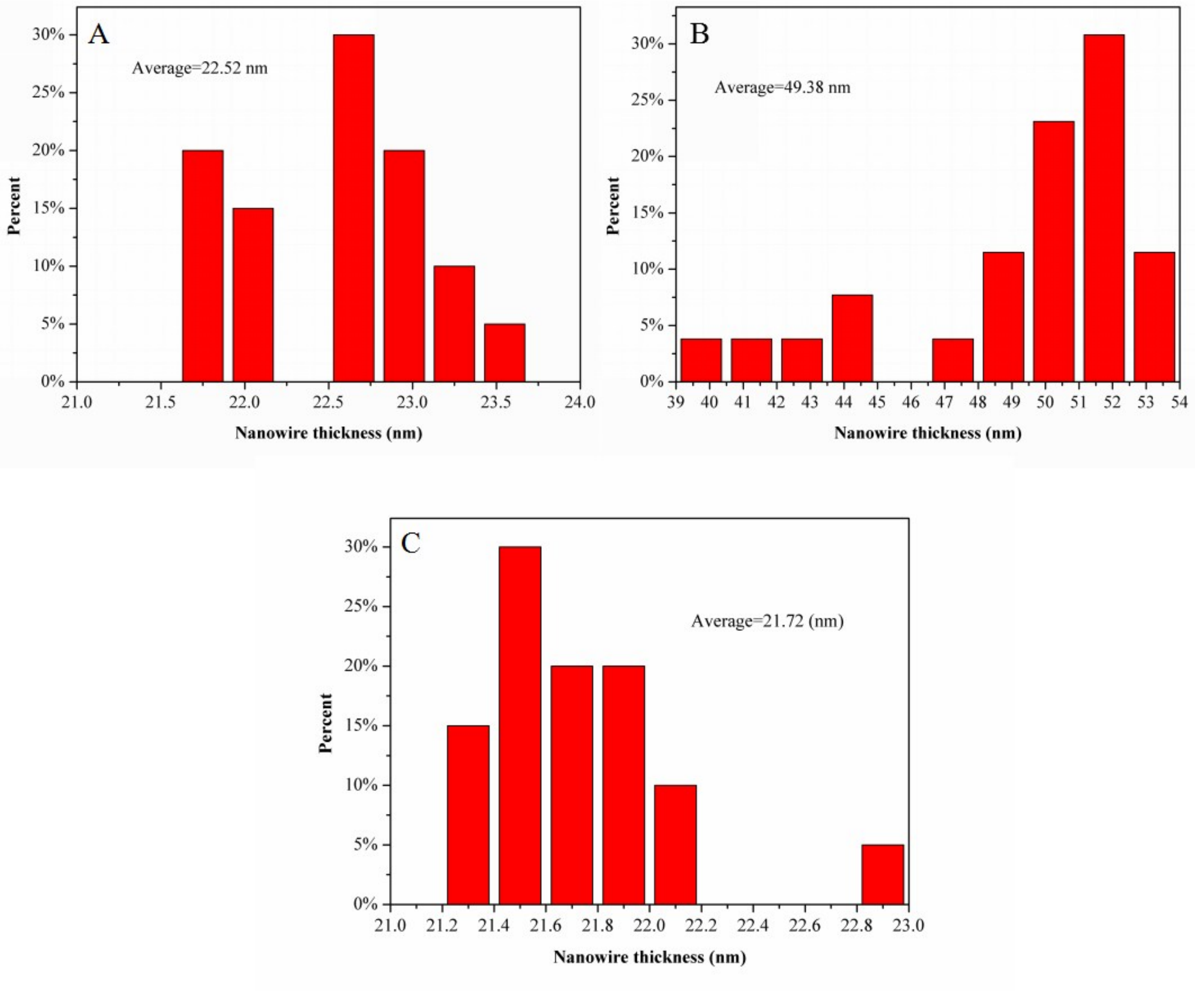

Figure S1. Size distribution of (A) MnO_2 nanowires (B) MnO_2/PPy and (C) MnO_2 in MnO_2/PPy .

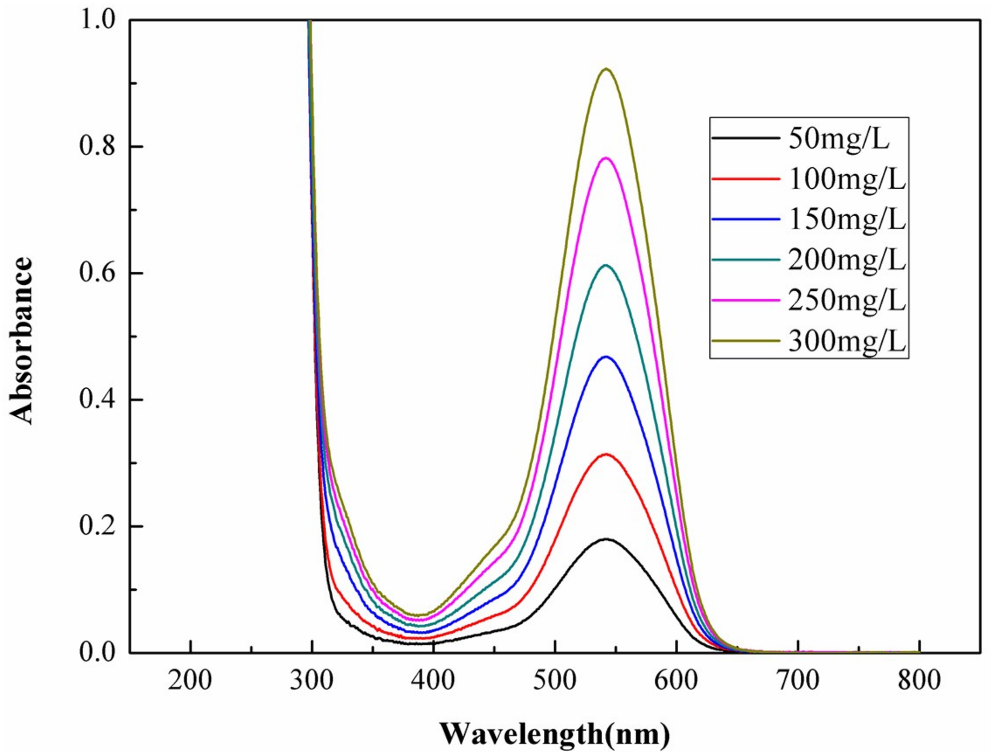

Figure S2. The absorbance of hexavalent chromium ion in different concentrations.

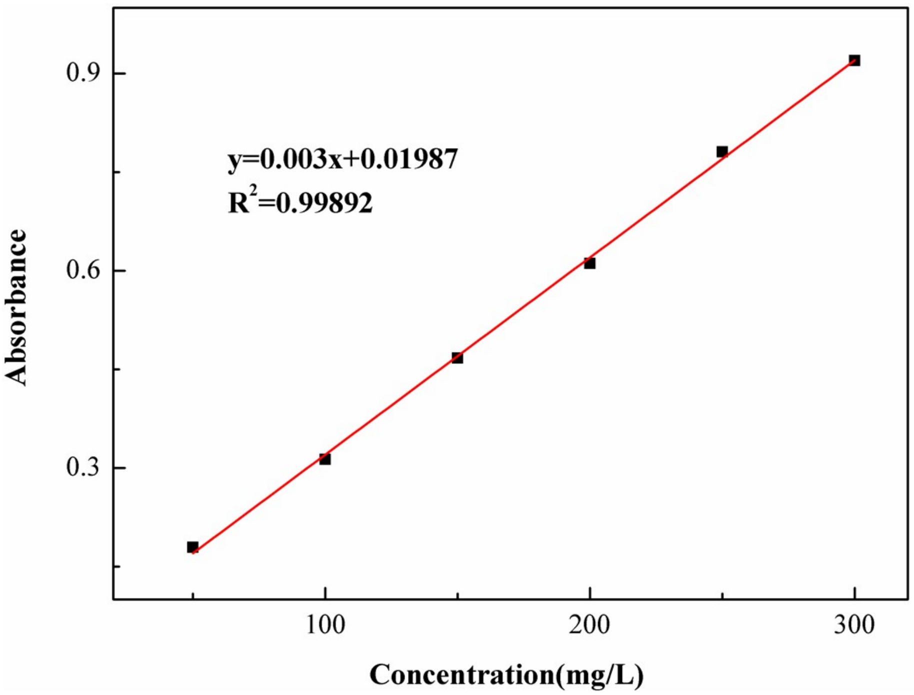

Figure S3. The linear relationship between the concentration and absorbance.

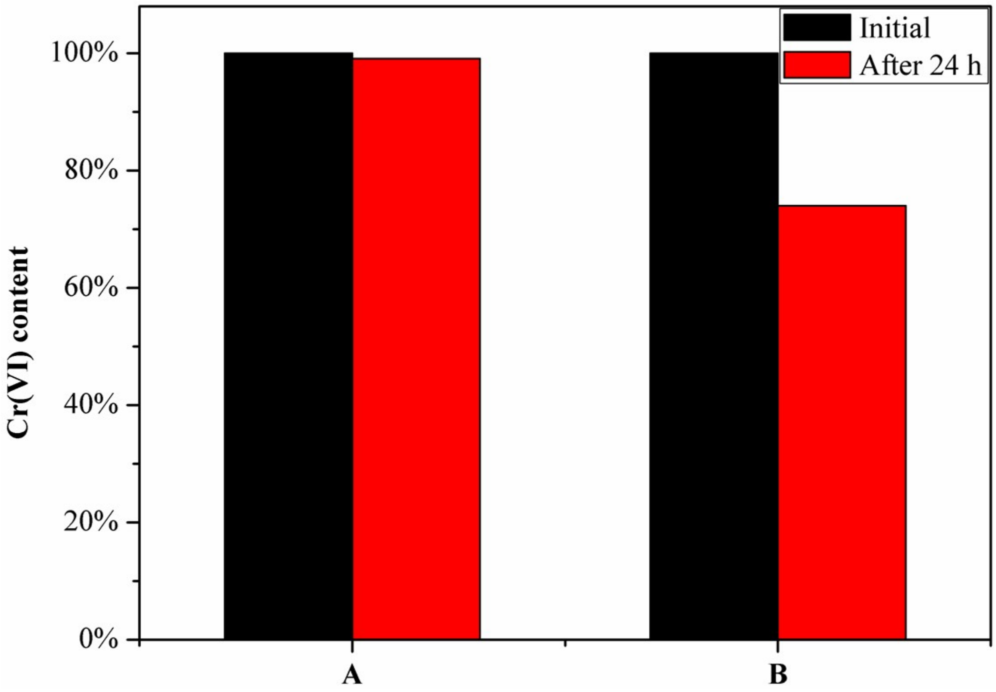

Figure S4. Controlled experiments about (A) without use of GMFP in the presence of acid (pH=2) and (B) without acid only use GMFP (Cr(VI) solution diluted with deionized water, 250 mg/L).

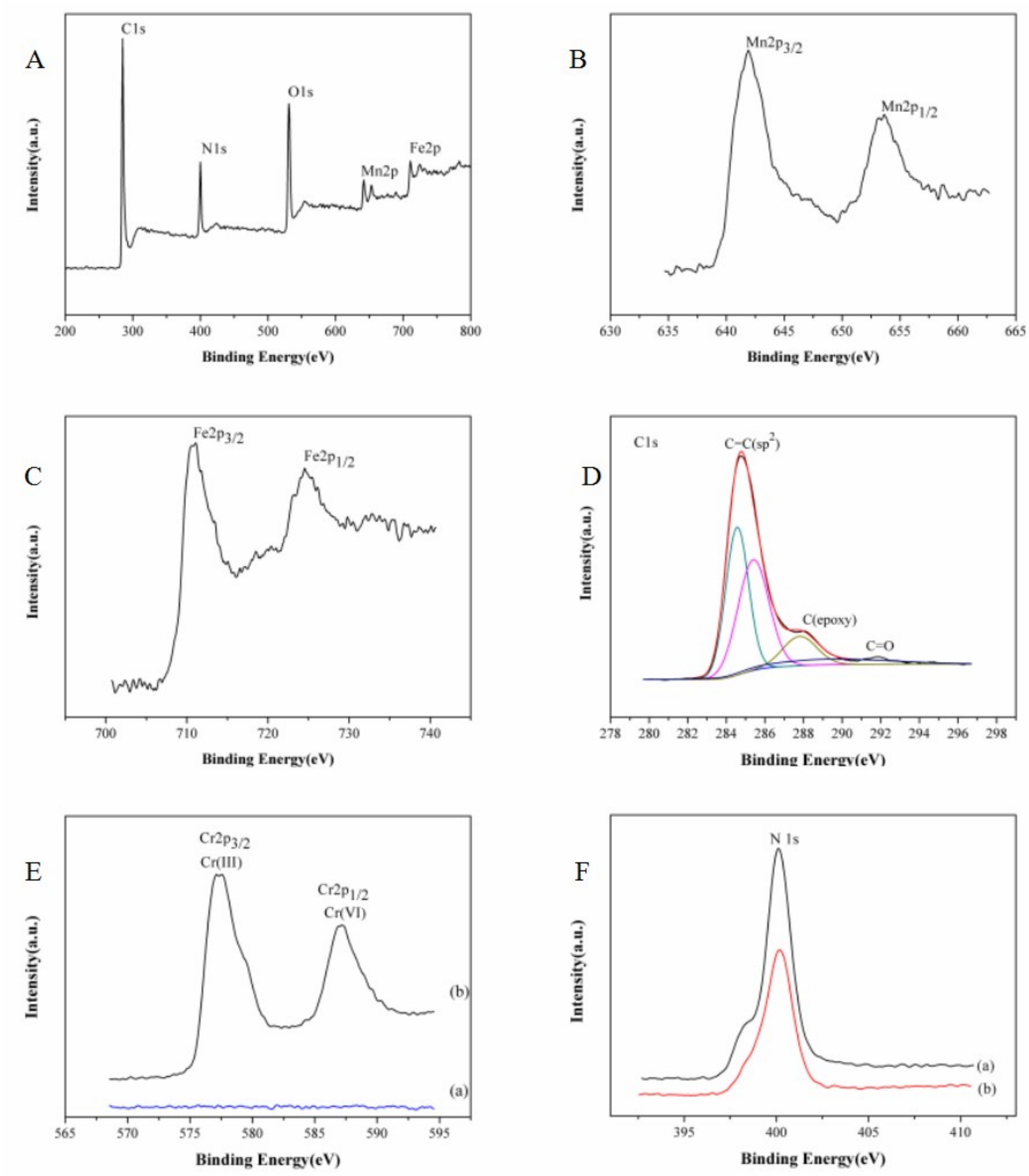

Figure S5. (A) Full range XPS spectra of GMFP; (B-D) XPS spectra of Mn2p, Fe2p, and C1s of GMFP; (E) XPS spectra of Cr2p of GMFP before (a) and after (b) adsorption of Cr(VI) ions ; (F) XPS spectrum of N1s of GMFP before (a) and after (b) adsorption of Cr(VI) ions.

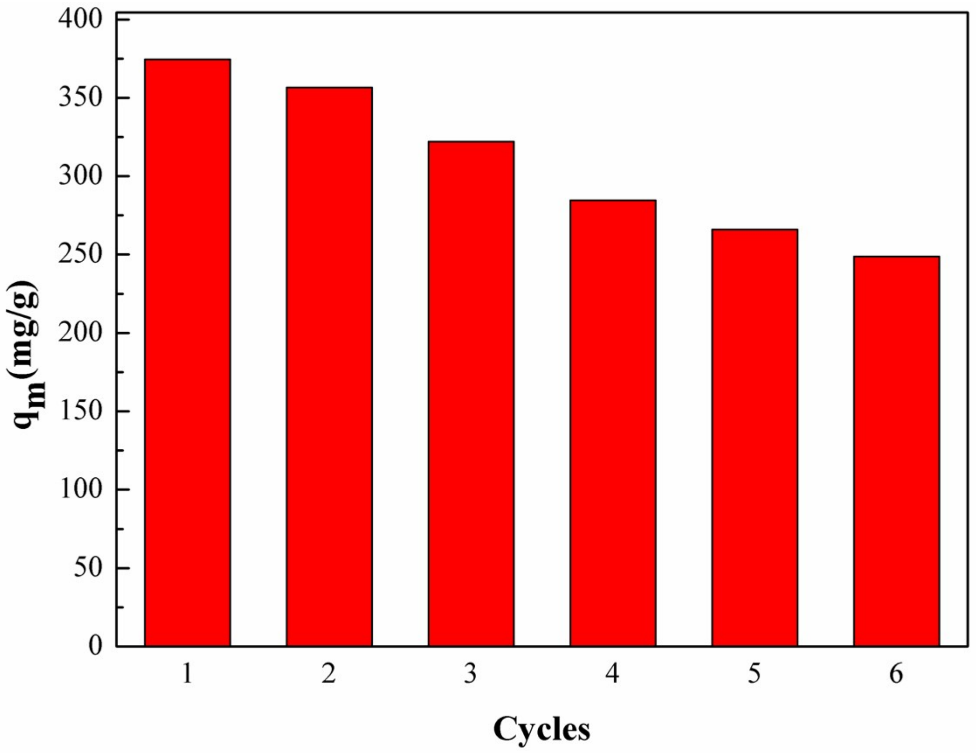

Figure S6. Reusability of GMFP for chromium ion adsorption.

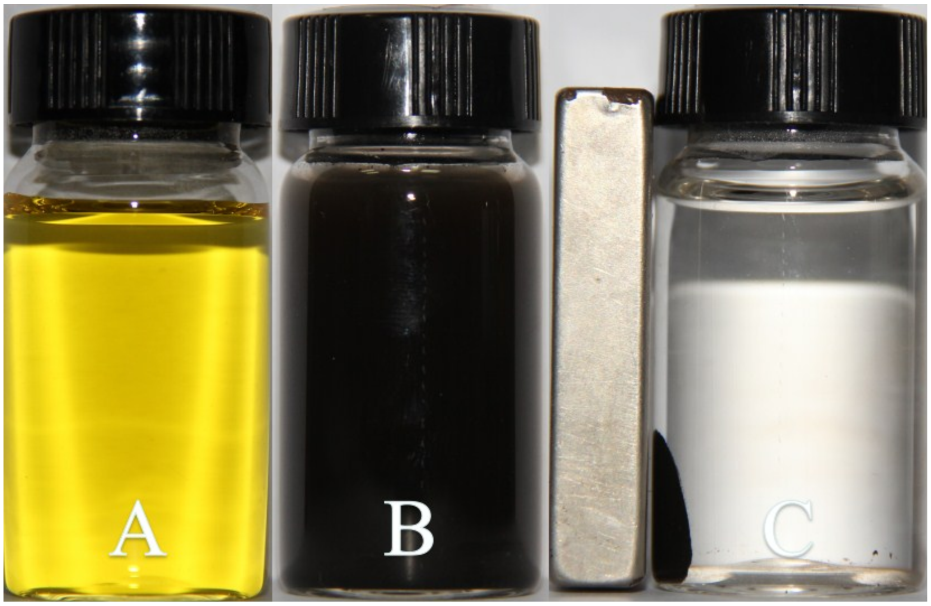

Figure S7. (A) The photograph of hexavalent chromium ion (100mg/L) (B) GMFP nanocomposites can be dispersed in water and (C) separated from water using a magnet .

REFERENCES

(1) Guo, X.; Fei, G. T.; Su, H.; Zhang, L. D. High-Performance and Reproducible Polyaniline Nanowire/Tubes for Removal of Cr(VI) in Aqueous Solution. *J. Phys. Chem. C* 2011, 115, 1608-1613.

(2) Wang, D.; Zhang, G.; Zhou, L.; Wang, M.; Cai, D. Synthesis of a multifunctional graphene oxide-based magnetic nanocomposite for efficient removal of Cr (VI). *Langmuir*, 2017, 33 (28), 7007-7014.

(3) Wang, L.; Bao, J.; Wang, L.; Zhang, F. One-Pot Synthesis and Bioapplication of Amine-Functionalized Magnetite Nanoparticles and Hollow Nanospheres. *Chem-A. Eur. J.* 2006, 12 (24), 6341-6347.

(4) Luo, C.; Tian, Z.; Yang, B.; Zhang, L.; Yan, S. Manganese dioxide/iron oxide/acid oxidized multi-walled carbon nanotube magnetic nanocomposite for enhanced hexavalent chromium removal. *Chem. Eng. J.* 2013, 234, 256-265.

(5) Anupam, K.; Dutta, S.; Bhattacharjee, C.; Datta, S. Adsorptive removal of chromium (VI) from aqueous solution over powdered activated carbon: Optimisation through response surface methodology. *Chem. Eng. J.* 2011, 173 (1), 135-143.
